# Do people have differing motivations for participating in a stated-preference study? Results from a latent-class analysis

**DOI:** 10.1186/s12911-021-01412-1

**Published:** 2021-02-06

**Authors:** Ilene L. Hollin, Ellen Janssen, Marcella A. Kelley, John F. P. Bridges

**Affiliations:** 1 Department of Health Services Administration and Policy, Temple University College of Public Health, Ritter Annex, 1301 Cecil B. Moore Ave Rm. 537, Philadelphia, PA 19122 USA; 2 Department of Health Policy and Management, Johns Hopkins Bloomberg School of Public Health, Baltimore, MD USA; 3 Department of Pharmaceutical and Health Economics, School of Pharmacy, University of Southern California, Los Angeles, CA USA; 4 Leonard D. Schaeffer Center for Health Policy and Economics, University of Southern California, Los Angeles, CA USA; 5 Department of Biomedical Informatics, The Ohio State University College of Medicine, Columbus, OH USA; 6 Department of Health Behavior and Society, Johns Hopkins Bloomberg School of Public Health, Baltimore, MD USA

**Keywords:** Stated preferences, Surveys, Discrete choice experiments

## Abstract

**Background:**

Researchers and policy makers have long suspected that people have differing, and potentially nefarious, motivations for participating in stated-preference studies such as discrete-choice experiments (DCE). While anecdotes and theories exist on why people participate in surveys, there is a paucity of evidence exploring variation in preferences for participating in stated-preference studies.

**Methods:**

We used a DCE to estimate preferences for participating in preference research among an online survey panel sample. Preferences for the characteristics of a study to be conducted at a local hospital were assessed across five attributes (validity, relevance, bias, burden, time and payment) and described across three levels using a starring system. A D-efficient experimental design was used to construct three blocks of 12 choice tasks with two profiles each. Respondents were also asked about factors that motivated their choices. Mixed logistic regression was used to analyze the aggregate sample and latent class analysis identified segments of respondents.

**Results:**

629 respondents completed the experiment. In aggregate “study validity” was most important. Latent class results identified two segments based on underlying motivations: a quality-focused segment (76%) who focused most on validity, relevance, and bias and a convenience-focused segment (24%) who focused most on reimbursement and time. Quality-focused respondents spent more time completing the survey (*p* < 0.001) and were more likely to identify data quality (*p* < 0.01) and societal well-being (*p* < 0.01) as motivations to participate.

**Conclusions:**

This information can be used to better understand variability in motivations to participate in stated-preference surveys and the impact of motivations on response quality.

## Background

Patient preference information can be elicited via a variety of stated-preference methods, including discrete choice experiments (DCEs), to assess patient (or caregiver) priorities and preferences for treatment or health state attributes [1]. Stated-preference methods are increasingly used in a variety of clinical and policy-related contexts such as clinical decision-making, drug and device development, regulatory review, and clinical trial design [2, 3]. Fundamental to the face validity of studies using stated-preference methods is the assumption that the survey respondents express their true preferences for profiles [1]. The degree to which this assumption is met is an issue of response quality and represents an important methodological issue in survey research in general and in stated-preference method research in particular [4–6].

Experimental and social psychology research has found the rates of careless or insufficient effort by respondents to be 8–12% [7]. Survey research occurring in low-stake settings is susceptible to low response quality [8, 9]. Stated-preference research using methodologies like choice-based conjoint analysis and DCEs are particularly susceptible to low response quality for multiple reasons. First, the methods are complex and increased complexity is associated with increased attribute non-attendance [10]. As such methodological research advancing the use of DCEs in health preference research has focused on instrument quality issues such as survey design, content development, and experimental method design or analytic methods [4, 11–17]. These factors contribute to minimizing the possibility of invalid data due to lack of relevancy, omitted variable bias, misunderstanding or misrepresentation. While a high-quality DCE instrument accompanied by vigorous analytic methods is necessary for valid, high quality data output, it is not sufficient. Second, DCEs have high cognitive burden due to their complexity and repetitive nature. They also often require learning at the beginning and respondents experience fatigue at the end [18]. The inclusion of too many choice tasks can increase this cognitive burden but this upper limit of choice tasks remains unknown [19]. As a result, respondents may have difficulty comparing alternatives or making trade-offs and instead focus solely on one attribute that they consider to be the most important. Third, DCEs often ask respondents to express preferences for hypothetical (non-market) goods and services and therefore are especially low-stakes. Use of large, online general population panels may further contribute to this.

This objective of this study was to measure what respondent preferences are for hypothetical preference studies about diabetes and identify segments of respondents based on self-reported differing underlying motivations. The effect of these motivations on stated-preference survey estimates and response quality is an important methodological question that remains largely unknown [20]. An understanding of respondent motivations, and their impact on survey results, will help ensure confidence in results of these stated-preferences studies as the methodology continues to grow in healthcare. We use this information to better understand response quality in stated-preference studies that inform decision-making.

## Methods

Response quality is a reflection of how closely stated preferences in the study reflect actual preferences. It is a function of both task complexity and respondent characteristics, but characteristics that have been studied tend to focus on demographic and socioeconomic variables that are expected to be related to cognitive ability [21]. For instance, lower education and advanced age are hypothesized to be associated with lower cognitive ability and poor consistency. Response quality may also depend on motivations, which are distinct from respondent characteristics, although there may be a relationship between the two. For instance, if income is associated with higher opportunity cost of time, it could be inversely related to motivation and response quality [21]. Little is known about motivations to respond to stated-preference surveys, however drawing on a conceptual framework for survey research categorizes respondents as either extrinsically or intrinsically motivated [8]. Response quality for extrinsically motivated individuals may be a function of incentives, both monetary and non-monetary, whereas response quality for the intrinsically motivated individuals may be a function of enjoyment, curiosity, or desire to voice their opinion [8].

### Survey design

The survey was a follow-on study to a randomized study comparing different ways patients with diabetes evaluate their preferences and priorities [17, 22–24]. The results of that study were discussed among a diverse group of experts through a Diabetes Action Board (DAB) and a day-long workshop. The DAB (n = 29) included individuals with experience in stated-preference research from industry, regulatory agencies, funding agencies, diabetes patient advocacy groups and academic institutions. Qualitative findings from those discussions determined the need for a follow-on study aimed to assess the relevance of stated-preference methods to patients and stakeholders [4].

For this survey, respondents were asked about their opinion on the characteristics of a survey study in the context of a study in a local hospital to learn about the preferences of its patients. Through qualitative engagement stakeholders identified eight qualities of stated-preference studies that were desired from their perspective. These included aspects of understanding/interpretation by respondents, relevance to stakeholders, using methods that match the research question, external validity, using diverse samples, transparency of methods, internal validity, and patient/population centeredness [4]. These concepts were adapted into attributes that might affect an individual’s decision to participate in a survey.

The survey was administered through GfK Knowledge Panel, a nationally representative online panel that can oversample for race and ethnicity (African Americans and Hispanics). The sample was drawn from a random sample of the general United States population who had not participated in the study before and a random sample of the 1,103 original participants who were patients with diabetes. The combination of these samples was used to limit the concern that the use of large online samples comprised of participants that do not have the condition being studied would introduce quality issues and to evaluate the generalizability of the findings from the diabetes patient sample to the general population. The survey collected self-reported demographic data, clinical information and preference data. Respondents were compensated for their time with a $10 (USD) cash equivalent.

Preference data for this study was collected using a discrete choice experiment (DCE). The experimental design was a D-efficient design (with zero priors) with three blocks of 12 tasks developed by Ngene (ChoiceMetrics, 2012) [25]. The choice for a zero prior design was made to maximize orthogonality and because we did not have empirical data (just assumptions) on the direction of preferences [12]. Each task presented two profiles, each with 5 attributes and 3 levels.

### Survey content

Attributes were developed using the literature as previous work has laid out the importance of these attributes in DCEs, either by expressing their importance in terms of ways to avoid them, how they may contribute to limitations of a study, or through the development of tools to measure these in DCEs [4, 26, 27].

The attributes of the studies represented six qualities of patient preference studies: validity, relevance, bias, burden, time and payment. Each attribute had 3 levels. For four attributes (validity, relevance, bias and burden) the levels were low, medium or high. When displayed to respondents, levels were shown with stars to remind respondents what level is considered best by researchers [28]. For instance, five stars were shown with high levels for validity and relevance, but five stars were shown with low levels for bias and burden. The levels for the time attribute were 15, 30, or 45 min and the levels for the payment attribute were $0, $25, or $50 (USD). Each attribute was defined for the respondent (Table 1).

**Table 1:** Attributes, definitions, levels used in the discrete choice experiment and aggregate results of discrete choice experiment

Attribute	Definition	Levels	Odds Ratio^a^	95% CI	References
Validity	A study is valid if the preferences it measures are the same as the preferences that people have in the real world	Low	2.4	(2.2, 2.6)	[4, 26]
		Med			
		High			
Relevance	A study needs to measure preferences that are relevant to patients and the disease it studies	Low	1.8	(1.7, 1.9)	[26, 27]
		Med			
		High			
Bias	A study should not try to influence people’s responses by pushing people to answer in a specific way. A biased study might not measure actual preferences	Low	1.7	(1.6, 1.8)	[26]
		Med			
		High			
Burden	A study can be easy or difficult to complete. This is related to the number of questions and the number and types of characteristics people have to think about	Low	1.2	(1.1, 1.3)	[26, 27]
		Med			
		High			
Time	How long it takes to complete a preference study	15 min	1.1	(1.1, 1.2)	[27]
		30 min			
		45 min			
Payment	How much people get paid to complete a preference study in USD	$0	1.4	(1.3, 1.6)	[27]
		$25			
		$50			

*CI *confidence interval; *min *minutes; *med *medium

^a^Odds ratio is for a one-level change in attribute

To pace respondents and ensure understanding of the attributes that represented concepts likely to be less familiar to participants (validity, relevance, bias and burden), they were prompted to answer a question about what level of the attribute researchers would aim to achieve. For instance, after reading a definition of validity, respondents were asked, “What level of validity do you think researchers try to achieve?” If respondents selected an answer other than “high” they were prompted with additional explanation as to why researchers would like their study to have high validity.

**Fig. 1:**
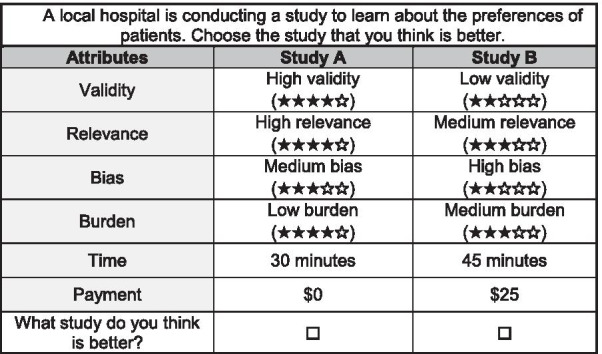
Sample task from discrete choice experiment

For the experiment, respondents viewed 12 tasks in which they were shown the characteristics of two different preference studies and asked to select the study that the respondent thought was a better study. An example task is shown in Fig. 1.

Following the preference experiment, respondents were asked about the factors that motivated their choices. They were asked “What things did you keep in mind when choosing between studies?” They selected all factors that applied from a closed-ended, pre-defined list that included 8 potentially motivating factors (completion rate, decision making, respondent burden, research results, patient benefit, society’s well-being, scientific publication, community centered). This list was defined from previous community engagement efforts [29].

### Analysis

The DCE was analyzed for the aggregate sample using mixed logit regression with an assumption that all independent variables were normally distributed for model succinctness [30]. The estimated coefficients were converted to odds ratios. The utility function for the models was specified as:

$$Uijt= {\beta }_{1n}{\mathrm{Validity}}_{njt}+ {\beta }_{2n}{\mathrm{Relevance}}_{njt}+ {\beta }_{3n}{\mathrm{Bias}}_{njt}+ {\beta }_{4n}{\mathrm{Burden}}_{njt}+ {\beta }_{5n}{\mathrm{Time}}_{njt}+{\beta }_{6n}{\mathrm{Payment}}_{njt}+ {\varepsilon }_{njt}$$

where U is the utility (U) participant i acquires from choosing treatment j for task t, ɛ is participant-specific random error and incorporate both preference estimates and variance-scale for the respective treatment characteristics. Latent class analysis was then used to identify different types of respondents based on their observed choices [31]. The analysis was conducted using Stata's mixlogit choice command with the standard options of 50 Halton draws with independent coefficients. Choice was treated as a binary dependent variable. The profile attributes were the independent variables and coded as continuous variables where low/medium/high were coded in one-step increments for model simplicity and to allow comparability of preferences across classes [11]. Preference estimates for these variables can be interpreted as change in utility if the attribute changed from low to medium or medium to high. An alternative approach using the same model specification but with effects coded attributes was also explored.

Akaike Information Criterion (AIC) and Bayesian Information Criterion (BIC) are criteria used to select the best fitting model among a finite set of models for a given set of data [32]. We ran models and calculated AIC and BIC values for models with up to 8 classes. AIC and BIC decreased with each subsequent class indicating a better model fit for each additional class modelled (Additional file 1: Table A1). We examined the output for both the 2- and 3-class models to determine how results differ. We selected the 2-class model because interpretability is justified as one of the most important factors in selecting the model and because we did not find any compelling differences between the two models to justify the increased complexity from reporting three classes.

Class membership was assigned to respondents based on their probability of membership in that class. Characteristics of respondents likely to fall into each class were tested using t-tests for means and Chi-squared tests for categorical variables. We also ran logistic regression models with the assigned class membership as the dependent variable and participant characteristics as the independent variables. Analyses included all participants who completed at least one choice task [33, 34]. All analyses were conducted using Stata version 13.0 (StataCorp, College Station, Texas).

## Results

The final sample included 629 respondents. About half of the sample (50.1%) originated from the previous diabetes sample and the remaining (49.9%) were newly sampled from the random sample. Results were compared between those with and without diabetes using a Wald Test and a Swait and Louviere test and no differences were found [35]. This justified the pooling of the two samples.

The sample had a mean age of 56.5 years (SD = 16.8) and was split evenly between males (48.9%) and females. About half of the respondents were white (49.9%) and more than half achieved a high school degree as their highest level of educational attainment (58%). Compared to the general population, persons with diabetes were younger (mean age = 63.8 vs. 47.46, *p* < 0.001), more likely to be black (22.7% vs. 26.6%) and Hispanic (23.5% vs. 25%), making race statistically significantly different between the diabetes population and general population (*p* < 0.001). All results presented include both original sample participants and newly sampled participants.

### Preference results

#### Aggregate results

In the main mixed logit regression model, participants showed the strongest preferences for a one-step increase in study validity (odds ratio: 2.40, SE: 0.10) (Table 1) Preferences for the next most important attributes were similar; a one-step increase in relevance (1.79, SE: 0.06) and a one-step decrease in bias (1.73, SE: 0.05). The fourth most preferred attribute was a $25 USD increase in payment (1.44, SE: 0.07). The least preferred attributes were a one-step decrease in burden (1.21, SE: 0.03), and a 15-min decrease in time (1.11, SE: 0.03). The alternative model with effects coded attributes found the same ordering of preferences and full results from that model are available in an additional file (see Additional file 1: Table A2).

#### Results by class

**Fig. 2:**
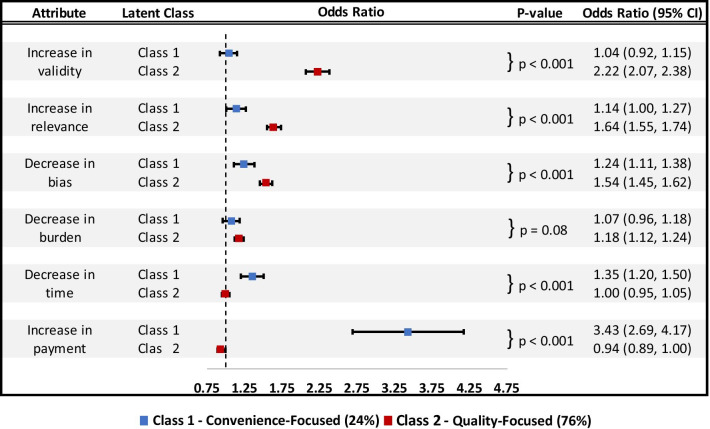
Latent class results of discrete choice experiments. *Note*: All changes represent a one-level change

Latent class analysis identified two classes of respondents with different preferences. Class membership was assigned based on the class to which the respondent had the highest probability of membership. The two classes differed significantly by age, education level, and diabetes prevalence (Table 2). Results from the logistic regression to identify the association between respondent characteristics and probability of class membership are available in Additional file 1: Table A3. Class 1 was the smaller class consisting of 24% of participants and showed strong preferences for participant reimbursement (Fig. 2). Class 2 was the larger class consisting of a large majority of respondents (74%) and they demonstrated strong preferences for study quality. Class 1 showed the strongest preferences for attributes related to study convenience: a $25 USD increase in payment (3.43; SE: 0.37) and a 15 min decrease (1.35; SE: 0.08). In contrast these two attributes were least preferred by class 2 (0.94; SE: 0.03 and 1.00; SE: 0.03). Class 2 most preferred increases in attributes related to study quality: increase in validity (2.22; SE: 0.08), increase in relevance (1.64; SE: 0.05), and decrease in bias (1.53; SE: 0.04). Preference weights from the discrete choice experiment and motivating factors by class with a 3-class model are available (see Additional file 1: Figs. A1 and A2).

**Table 2:** Demographic characteristics for respondents by class (n = 629)

	Class 1		Class 2		*p *value
	24%		76%		
	Freq (n = 148)	Percent 100%	Freq (n = 481)	Percent 100%	
Age, mean (SD)	52.97 (16.8)	–	57.52 (16.7)	–	0.004^a^
Male	64	43.2%	244	50.7%	0.110
Race/ethnicity					0.600
Black	36	24.3%	107	22.2%	
Hispanic	32	21.6%	116	24.1%	
Other	8	5.4%	16	3.3%	
White	72	48.6%	242	50.3%	
Education					0.025^a^
< High school	13	8.8%	34	7.1%	
High school	44	29.7%	122	25.4%	
Some college	56	37.8%	146	30.4%	
Bachelor's degree +	35	23.6%	179	37.2%	
Diabetes	71	48.0%	275	57.2%	0.049^a^

*Freq* frequency, *SD* standard deviation

^a^Statistically significant at the 0.05 level

**Fig. 3:**
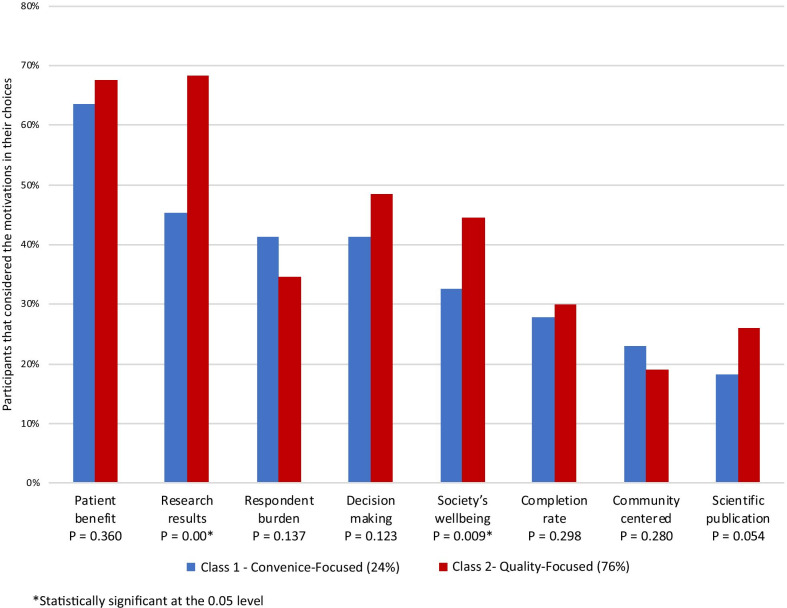
Motivating factors by class

Patient benefit motivated participants most in their decision making; 419 (67%) indicated that they considered this perspective (Fig. 3). 395 participants (63%) reported that they considered the quality of the preference data that could be obtained from the study. In comparing the two classes, quality of data was associated with people more likely to be in the quality-focused class than the convenience-focused class (68% vs. 45%; *p *value < 0.01). Similarly, society well-being was a motivation more likely to be reported in the quality-focused class vs. the convenience-focused class (44% vs. 32%; *p *value < 0.01). All other motivations were similar between the two classes.

**Fig. 4:**
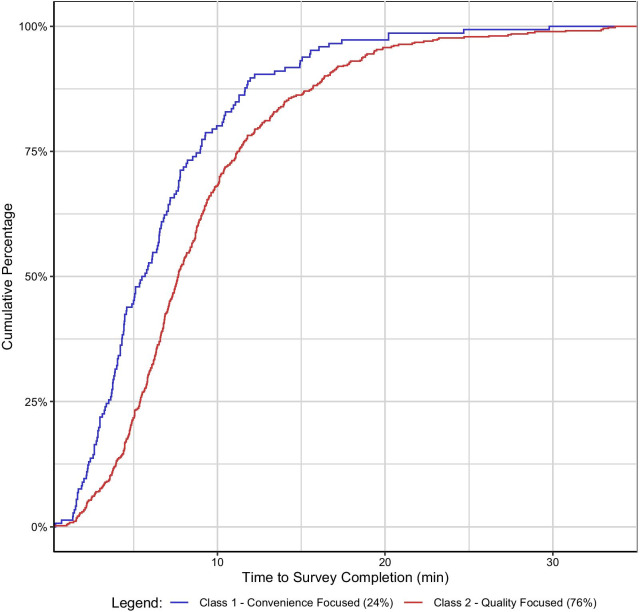
Cumulative percentage of respondent completion time

Finally, we analyzed the response time for the survey based on class membership. The average response time for convenience-focused respondents was statistically significantly different from the average time for quality-focused respondents (6.8 versus 9.0 min; *p *value < 0.001). Seventy-five percent of the convenience-focused class had finished the survey in under 9 min whereas seventy-five percent of the quality-focused class finished the survey in 11.2 min (Fig. 4).

## Discussion

Response quality remains an important, yet understudied issue in discrete choice methodology research. This study aims to understand what factors motivate patients and the general public to participate in DCEs and what respondents want to get out of this type of research. The majority of participants (76%) valued study quality, but about a quarter of respondents strongly valued convenience. While both segments had similar motivations, the quality-focused majority were more likely to be motivated by measuring real preferences and benefits to society. Understanding different response styles by motivation type can inform study design and selection of incentives aimed at increasing response rates.

These findings have implications for future stated-preference work. First, we demonstrate that motivations differ across people and those motivations may be discernable as part of screening process. A limitation of this finding is that it would likely require substantial resources to screen people based on these motivating factors. Attempts at identifying motivating factors that are fast and easy may be transparent to prospective respondents. More subtle attempts, such as through individual responses to screening questions, are costly and risk fatiguing respondents before they even get to the research under investigation by the DCE. A good next step would be to design and validate a brief questionnaire that would predict whether or not respondents are quality-focused or convenience-focused.

The discussion of screening possibilities assumes that the motivations are directly related to response quality, but that is not known. Next steps should include determining how motivations are related to response quality. The study methodology we used is a DCE, so all preferences are relative to one another, but the data do not speak to whether or not an attribute is unimportant. A convenience-focused respondent may prefer convenience relative to quality, but still value data quality and therefore their preference does not necessarily inhibit their ability to provide quality answers. Our analysis of response time shows that convenience focused people took less time to complete the survey. While it is often assumed that lower response time is associated with poor quality respondents, it could also be that convenience-focused people are more efficient survey takers.

Once we better understand how the motivation of respondents is related to response quality, a philosophical discussion is warranted about the trade-offs between representativeness versus bias and whether or not to exclude low quality responses from the primary analysis. If representativeness is the goal, the inclusion of both quality-focused and convenience-focused people is warranted. Assuming convenience-focused people have lower response quality, researchers may not be able to accurately measure their preferences. However, their inclusion would bias preference study results toward zero, therefore resulting in more conservative estimates of preference weights. If limiting bias is the goal, then perhaps health economists should be screening out based on motivating factors that are associated with poor quality or try to shield their results from poor response quality through the use of consistency tests [21]. The trouble with identifying this type of non-random error is that some people may exert sufficient effort, but not enough to consider them "true" responses [7]. Furthermore, post-hoc efforts to identify low quality responses waste valuable data collection resources.

We report on a 2-class model based on primary motivation for participation, but there may be more classes. Akaike Information Criterion (AIC) and Bayesian Information Criterion (BIC) values were calculated for models with up to 8 classes and AIC and BIC decreased with each additional class indicating a better model fit. The greatest decreases in AIC resulted from a move from a single-class model to a 2-class model, and from a 2-class model to a 3-class model, therefore prompting us to examine the output for both the 2- and 3-class models. The results from the 3-class model also identified a quality-focused class, as well as two convenience-focused classes. The two convenience-focused classes had overlapping preferences on all attributes except one; for one class the payment attribute dominated. In the 3-class model, class 1 and 2 are similar in terms of the percentage of participants that considered the majority of factors as motivation in their choice. The greatest difference between the two convenience-focused classes in the 3-class model was that one class had a greater percentage motivated by respondent burden. Best practices encourage that the number of classes be chosen to address the underlying research question and ease of interpretation [11]. We ultimately favored a 2-class model in order to keep the results actionable. The greater the number of classes, the less interpretable and actionable the results become. We did not find compelling differences between the two models to justify the increased complexity from reporting three classes.

### Limitations

This study has several limitations. First, this study presents a limited view of respondent motivations. We ask about eight motivations, but respondents may be motivated by many additional things. The benefit of including a limited number of motivations is decreased response burden. We did not include any free text fields for respondents to further explain their motivations or add additional motivations not listed. Likewise, we chose a limited set of attributes for our choice experiment.

Second, there may be limitations around the framing of the study. We asked about preferences for a study during a study. This may have resulted in respondents conflating preferences and motivations in general with how they felt at the time of the survey. For example, if a respondent disliked the study underway and felt it was a waste of time, they may have been more likely to answer the choices in a convenience-focused way. Furthermore, we framed the questions in terms of a general study at a hospital. If the study had been framed using a more concrete example (e.g., a specific condition or a specific decision), results may have differed. However, while it is reasonable to think that the proportion of respondents that are convenience-focused would be smaller in a DCE that measures preferences of patients affected by a particular condition, using a concrete example among a general sample would likely not have changed the results.

Finally, this study is susceptible to limitations that impact all DCEs. The model is sensitive to the attributes and levels chosen. The same study repeated with different definitions for the attributes or different levels could produce different results. The latent class analysis, while accounting for differences across classes, does not account for heterogeneity within classes. The model also assumes that all respondents and all tasks measure preferences equally well or equally poorly. However, given that the topic of this study is that respondent motivation may impact response quality, it would be remiss not to acknowledge that the same potential issue applies to this study.

## Conclusions

The need to better understand response quality for stated-preference survey methods is an important methodological question. This study measured what respondent preferences are for hypothetical patient preference studies conducted in a hospital setting. Participants showed the strongest preferences for a one-step increase in study validity, but segments of respondents based on differing underlying motivations were also identified. About one quarter of respondents were convenience-focused, preferring reimbursement and about three quarters of respondents were quality-focused preferring study quality. On average, quality-focused respondents spent more time completing the survey and were more likely to identify data quality and society well-being as motivations. This information can be used to better understand response quality in stated-preference studies.

## Supplementary Information

**Additional file 1.** Additional file contains the following supplementary exhibits: Tables A1, A2, and A3, and Figures A1 and A2.

## Data Availability

The datasets used and/or analyzed during the current study are available from the corresponding author on reasonable request.

## Abbreviations

AIC: Akaike information criterion
BIC: Bayesian information criterion
DAB: Diabetes Action Board
DCE: Discrete choice experiment
