# Supplementary material for: Do people have differing motivations for participating in a stated-preference study? Results from a latent-class analysis
Source: BMC Med Inform Decis Mak. 2021 Feb 6;21:44. doi: 10.1186/s12911-021-01412-1 (PMC7868025; doi:10.1186/s12911-021-01412-1)
Supplement: Supplementary file 1 — Additional file 1. Additional file contains the following supplementary exhibits: Tables A1, A2, and A3, and Figures A1 and A2. [file 12911_2021_1412_MOESM1_ESM.docx]

Additional File 1

**Table A1** Akaike Information Criterion (AIC) and Bayesian Information Criterion (BIC) statistics for continuous model

| **Number of Classes** | **Log-Likelihood** | **AIC** | **BIC** | **AIC Differences** |
| --- | --- | --- | --- | --- |
| 1 | -4267.8 | 8547.6 | 8593.3 | n/a |
| 2 | -3959.7 | 7919.4 | 7919.4 | 628.3 |
| 3 | -3757.5 | 7514.9 | 7514.9 | 404.4 |
| 4 | -3705.0 | 7410.0 | 7410.0 | 104.9 |
| 5 | -3593.3 | 7186.6 | 7186.6 | 223.4 |
| 6 | -3561.2 | 7122.5 | 7122.5 | 64.1 |
| 7 | -3536.4 | 7072.8 | 7072.8 | 49.7 |
| 8 | -3529.6 | 7059.2 | 7059.2 | 13.6 |

**Table A2** Aggregate results of discrete choice experiment using attributes with effects coding

| **Attribute** | **Definition** | **Level** | **Coefficient** | **95% CI** |
| --- | --- | --- | --- | --- |
| **Validity** | A study is valid if the preferences it measures are the same as the preferences that people have in the real world. | Low | -0.984*** | (-1.081, -0.888) |
|  |  | Medium | 0.095*** | (0.039, 0.151) |
|  |  | High | 0.889*** | (0.794, 0.984) |
| **Relevance** | A study needs to measure preferences that are relevant to patients and the disease it studies. | Low | -0.690*** | (-0.768, -0.611) |
|  |  | Medium | 0.098*** | (0.039, 0.157) |
|  |  | High | 0.592*** | (0.513, 0.670) |
| **Bias** | A study should not try to influence people’s responses by pushing people to answer in a specific way. A biased study might not measure actual preferences. | Low | 0.460*** | (0.388, 0.531) |
|  |  | Medium | 0.171*** | (0.106, 0.235) |
|  |  | High | -0.631*** | (-0.705, -0.557) |
| **Burden** | A study can be easy or difficult to complete. This is related to the number of questions and the number and types of characteristics people have to think about. | Low | 0.197*** | (0.133, 0.261) |
|  |  | Medium | 0.024 | (-0.032, 0.080) |
|  |  | High | -0.221*** | (-0.283, -0.159) |
| **Time** | How long it takes to complete a preference study | 15 min | 0.071** | (0.008, 0.134) |
|  |  | 30 min | 0.061* | (-0.003, 0.124) |
|  |  | 45 min | -0.132** | (-0.196, -0.067) |
| **Payment** | How much people get paid to complete a preference study in USD | $0 | -0.473*** | (-0.574, -0.373) |
|  |  | $25 | 0.156*** | (0.094, 0.219) |
|  |  | $50 | 0.317*** | (0.217, 0.417) |

*** p<0.01, ** p<0.05, * p<0.1; P-values for omitted categories were calculated using the formula put forth in Altman, 2011; CI=confidence interval; min=minutes

**Reference**

Altman DG, Bland JM. How to obtain the P value from a confidence interval. BMJ. 2011;343: d2304.

**Table A3** Logistic regression results to identify association between respondent characteristics and probability of class 2 membership (n=629)

|  | **Coefficient** | **Standard Error** | **P-value** |
| --- | --- | --- | --- |
|  |  |  |  |
| Age | 0.014 | 0.007 | 0.035 |
| Male | 0.262 | 0.194 | 0.176 |
| Race/ethnicity |  |  |  |
| White | 0.491 | 0.468 | 0.294 |
| Black | 0.424 | 0.492 | 0.389 |
| Hispanic | 0.699 | 0.495 | 0.158 |
| Education |  |  |  |
| Some College | -0.104 | 0.226 | 0.644 |
| Bachelor's degree | 0.609 | 0.245 | 0.013 |
| Diabetes | 0.096 | 0.225 | 0.669 |

**Figure A1.** Results of the discrete choice experiment with a 3-class model

**^+^**The reported p-values are the result of pairwise comparisons across classes within each attribute. The p-value comparing Class 1 to Class 2 is in the first row, Class 2 to Class 3 is in the second row, and Class 1 to Class 3 is in the third row of each attribute.

**Figure A2**. Motivating factors by class in the 3-class model
